# Review of the Conservative Treatment of the Dental Pulp

**Published:** 1879-10

**Authors:** Louis Jack

**Affiliations:** Philadelphia


### REVIEW OF THE CONSERVATIVE TREATMENT OF THE DENTAL PULP.

BY LOUIS JACK, D. D. S., PHILADELPHIA.

*Read before the Pennsylvania State Dental Society.*

When this subject was selected for presentation to this Association, it was the intention to make a resume of whatever had been attempted in the nature of protective treatment of the dental pulp, as described at different times in the current dental publications. This has been rendered unnecessary by the appearance in the Transactions of the New York Odontological Society for the year 1877, of an essay by Dr. S. Morgan Howe, which covers the various methods from the earliest times.

It may be instructive, however, to classify the different methods of treatment which have been practiced, and to define the defects and advantages of each. They may be divided into a. The Mechanical.
b. The Chemical.
c. The Therapeutical.

### MECHANICAL TREATMENT.

This may include the various attempts of what was designated by the Dentists of the earlier part of the present century as "capping the nerve."

It consisted in so applying over the pulp, an arch covering of lead or gold, in such a manner that no portion of the cap should touch it. It was intended that the air-space included between the metal and the sensitive part, should prevent the irritation of Thermal alternations; it being believed at that time, that the pulp would not tolerate the contact of any substance.

Under this head may be included the almost impracticable method described by Dr. C. A. Harris, so frequently alluded to, and for which he claimed the most satisfactory results.

This was a simple arching of the gold foil over the exposed pulp during the process of packing. This plan does not differ essentially from the then commonly practiced method of capping. Capping the pulp in those days, it would appear, was effected (whatever the pretensions may have been), to enable the cavity in the tooth to be filled at once without immediate pain, and without sufficient consideration of the future interests of the patient.

The results of mere mechanical treatment at length became so unsatisfactory as to have led to a reverse practice—devitalization of the pulp—which at length became general, and was practiced without much question until within a few years.

The causes of the failures in arching would appear to be attributable either to the absence or inefficiency of therapeutic and chemical management, and the presence of the air-space.

The chemical treatment of that time, consisted in the employment of the actual cautery and the topical use of kreosote; the therapeutical, of tincture of opii and tannic acid, neither, excepting the kreosote, being indicated or useful. The air-space would inevitably become filled with serum, blood, or degenerated lymph, the presence of either being followed by decomposition, the resultant gases, causing a recurrence of serious pain.

### CHEMICAL TREATMENT.

Namely: the applications of pure carbolic acid, kreosote and oxychloride of zinc.

The reported results of this recent method, have thus far been so various and conflicting, that it is a difficult task to form a satisfactory conclusion from the opposing statements, some declaring universal success, and others universal failures.

The defect of chemical treatment or the application of drugs of sufficient strength to produce toxic effects, may be stated to be the undue irritation excited thereby, as frequently considerable portions of the pulp substance is devitalized, and often the whole organ is destroyed. It has become my conclusion, as a result of repeated trials, and of my observation of the attempts of others, that chemicals in full strength, especially such as have affinity for any of the elements of the pulp, are inapplicable, except as a last resort in cases where there already is disorganization of the exposed surface.

The first appearance of chemical treatment was the application of the actual cautery. The improbability of securing an actual white heat of an instrument so small as to be brought in contact with an exposed pulp, and the difficulty of making the application to many positions, rendered the actual cautery a mere probability, and often a simple pretence of treatment. Nothing having the appearance of intelligent chemical treatment appeared in our literature until the previous decade, when the rational use of kreosote, the employment of carbolic acid, which had then been recently introduced, and the application of oxy-chloride of zinc came into use. The physical effects of these are similar, or the same as the effects of the actual cautery, mainly to co-agulate the albumen of the tissue, thus producing, as claimed, a protective film of tissue.

### THERAPEUTICAL TREATMENT.

It may be defined that applications of remedies of such dilution or of such qualities as to be non-irritating, and possessing tonic or sedative powers, may be designated as therapeutic or healing to the pulp.

This class of remedies have been more used lately than formerly, and further experience promises to increase the number, and to enlarge the range of them. At present they are few, viz: aconitum, calendula officinalis, camphora, hypophosphate of lime, lacto-phosphate of lime and Canada balsam. To these may be added thymol and kreosote or carbolic acid, always in solution. Frequent reports of successful treatment have been made of all these remedies and it may be said a re-action has occurred towards reliance upon medicaments which do not excite irritation.

The accumulation of carefully recorded experience of conservative treatment of the dental pulp, has not been sufficient to enable a clear view of the whole subject to be attained.

A mode of treatment which would be applicable to one class of cases, and which will be followed by satisfactory results, may be utterly useless in another class; a distinction, the force of which an attempt will be made to illustrate in what shall follow.

It appears from the different discussions which have taken place on this subject, that some who do not hesitate to treat all simple non-painful cases of exposure, have no confidence in similar treatment in cases which have been attended by severe symptoms. Others attempt treatment in all cases. It will readily been seen that the proportion of success to failure by the former, will be greater than obtains in the practice of the latter.

It may, however, be stated as a safe deduction from the concurrent experience of a great many operators, that the treatment of the pulp is often followed by satisfactory reparations, and that it is desirable to extend a knowledge of the means of securing so beneficial an end. It is certainly not too much to expect, that at length the treatment of the denuded pulp will be as reliable as any class of operations in our specialty of surgery. Towards these purposes I shall now direct attention to the plan of treatment I have been practicing, believing that some of the features are of a valuable character.

The cases of disturbance of the pulp are properly divisible into the following classes:

Conditions depending upon the very close proximity of caries.

Accidental encroachments upon the pulp by cutting through the healthy dentine.

Conditions induced by the actual contact of caries at a small part; the pulp remaining covered by the gelatinous residue, and not previously the source of pain.

Conditions caused by the complete exposure to external irritants, as air, food, pressure, etc., and which have produced odontalgia.

Any extended description of these conditions it is unnecessary to make, and your attention can be more profitably occupied by the consideration of the treatment I have found most serviceable at my hands in each of the described conditions.

The treatment of cases of proximate exposure has been attracting much attention for several years, and deservedly so, for it may now be justly said, that there are more deaths of the pulp from the failure to recognize proximate exposures, and from disregard of the simple treatment required to preserve the pulp in these cases, than follow skillful conservative treatment of full exposures. The only safe plan to pursue in cavities at all near the pulp, and in all moderately deep cases of caries in the lateral incisors, central incisors and bicuspids, is to apply a small quantity of carbolic acid to the cavity, and then on the portion over the pulp, some non-ductor should be laid. In the small teeth above mentioned, my practice is, to touch the part nearest the pulp, with a solution of gum mastic, in either alcohol or ether, and in a moment the surface is perfectly protected.

In deeper cases I lay upon the mastic before it has hardened, a thin shaving of hard gutta percha. In the deepest cases I cover thickly the bottom of the cavity with either mastic or a solution of gutta percha in chloroform, and fill the deeper half of the cavity with oxy-chloride of zinc as a mechanical foundation.

It may be said here, that in the treatment of these, and of all pulp cases, it is indispensable to success that the rubber-dam be applied to effect complete control of moisture.

### ACCIDENTAL ENCROACHMENTS UPON THE PULP.

While there has always been danger of incautiously exposing the pulp by mistaken estimate of the depth of the cavity at a vulnerable point, or by the use of too much force in excavating; the risk has been much enhanced since the introduction of speeded drills and revolving excavators.

My method of treating these cases is to apply tincture of calendula, one part to four of water on a pledget of cotton, which is permitted to remain for a few minutes, when I cover the point of accidental exposure with gutta percha varnish, placing upon this a layer of gutta percha, one side of which is moistened in the varnish; or a disk of tin previously dipped in the varnish may be used. The cavity is then filled temporarily for a few days, and if no untoward symptoms occur, the cavity is then permanently filled. I do not remember having lost a single pulp in recent years when exposed in this way, and treated in this manner.

### CONDITIONS INDUCED BY ACTUAL CONTACT OF CARIES, BUT NOT SUBJECT TO ATTACK OF ODONTALGIA.

We now come to the treatment of the class of cases in which there has been the greatest disparity of views, and the most conflicting management. The method of some has been principally therapeutical; of others entirely chemical. My own disposition and practice has steadily leaned toward the former. It will not fail to be borne in mind in any consideration of the treatment of this class, that the pulp may have been exposed for many months, and in some cases for years without the patient having had an attack of toothache, or even of reflected pain; it would appear in such cases to be only necessary to remove the soft caries from the cavity, disinfect and neutralize the fluids of the remaining layer of caries and cover the bottom of the cavity without causing pressure, or using irritative medicaments. In such cases I employ carbolic acid, ten per cent. in glycerine to saturate the remaining layer of decay, first applying tincture aconitum and chloroform in equal parts. I then cut a piece of gutta-percha, fitting somewhat the bottom of the cavity, applying gutta percha varnish to the lower side, and then fill the remainder of the cavity with Hill's stopping or oxychloride of zinc, to be retained until there is assurance that the case may be permanently filled.

But often in the management of cases of this character, we may open the pulp to the air, or the patient may have experienced an evening or two of slight neuralgic pain on the side of the face on which is the afflicted tooth. Under these circumstances my practice is, after employing aconite, as before described, to cover the point of exposure with a paste composed of oxide of zinc and kreosote, as first described by Dr. Jas. S. King, in 1873; this, in turn, being covered either with a layer of oxy-chloride of zinc, or of sheet tin, to bear the pressure of the external filling. My success in this class of cases has been various, the majority of them, however, being successful. So many have been satisfactory, that I should consider it malpractice for an exposure of this class to be treated by devitalization.

The after-treatment consists in the application of tincture of aconitum 4 oz., chloroform 2 oz., to the gum over the affected tooth, which may be repeated on each successive day, if required. The necessity for the use of aconite in this manner is indicated, if the tooth becomes subject to any pain or is super-sensitive to cold applications.

The response to aconite in these conditions is generally prompt and effective, if properly administered. The application is made by means of a pledget of cotton as large as the end of the finger, saturated to excess, and the excess pressed out; this is then placed over the gum of the tooth as high as possible, and permitted to remain about a minute. To illustrate the power of aconite to control congestion of the pulp, it may not be unprofitable to report the case of a gunning accident to a boy in my practice. His gun had "kicked" him as he had termed it, breaking slightly the corners off the two centrals. The next day when he came in, the right tooth was decidedly pinkish in color from the injection of some of the disorganized blood corpuscles. Without much hope of saving his pulp, the gum was scarified, and aconite and chloroform applied. In two days the pinkishness had passed away; but in six months the tooth, while not discolored, was off-color slightly. After some months on excavating a small cavity which had come in the tooth, it was found to be excessively sensitive, which fact removed all apprehension of devitalization. The results of this case have always strengthened my previous exalted estimate of the value of aconite remedy when used for this purpose.

The permanent filling of these cases I defer until all disturbance has passed away. I have usually waited from two to five years, according to the apparent necessity before ingnfihtəshifillings. In some instances I have found secondary deposits of bone completely formed, hard and transparent in three years, which has been my shortest time for opening down to the pulp for examination of the old point of exposure. This, however, is a curiosity which I have abandoned as unnecessary and dangerous.

I have opened pulps apparently of this class which have remained for five years as comfortable as any tooth in the mouth of the patient, and by so doing, I have excited disturbances difficult to quell,

My present practice is to fill these cases permanently, after a fair trial of a year or more. This is done without removing the protective dressings over the pulp, which should be reinforced if they are not of a nature to bear the pressure which may be required in the performance of the operation.

### CONDITIONS CAUSED BY THE COMPLETE EXPOSURE TO EXTERNAL IRRITANTS, AND WHICH HAVE BEEN THE SOURCE OF REPEATED PAIN.

Those who have distinguished this class of cases from the simple non-painful ones, must have become aware of the difficult nature of their treatment.

While always presenting difficulties, they vary so much in the symptoms presented at first, and unexpectedly manifested during the course of the treatment, that it is almost impossible to lay down in an article so brief as this must be, a full and clear explanation of all the phases of management.

The unknown and not clearly ascertainable condition of the pulp, consequent upon the long exposure, the changes which in many cases have taken place in the blood vessels, the nerve fibres, the connective tissue, render the successful treatment of old cases exceedingly problematical.

A further experience of careful and persistent handling of all classes of exposures during the past six years, have not altered my views as expressed then in writing upon this subject. I have pleasure, however, in being able to state that the additional experience gained in that time, has enabled me to arrive at earlier conclusions, whether there is any hope to be entertained of success in any individual case. The changes in treatment during this period have saved my patient much disturbance, and have led to an increase in the per centage of recoveries of much diseased pulps.

It may be stated as a fundamental principle, that without a state of general health being enjoyed by the patient, it is futile to attempt any given case.

The influence of temperament is also an element of the largest importance, and should always enter into the prognosis. The nervo-sanguine being the most favorable temperament (always recuperative in its nature) and the bilio-lymphatic the most unfavorable temperamental conditions usually tending to chronic disturbances, and to the perpetuation of chronic states.

In order to make clear the application of the treatment to be pursued, of the pulps now under consideration, it is a necessary preliminary to make some inquiry of their physical condition, apart from changes which are not ascertainable at the time; such as the deposit of fat granules between the nerve fibres, and even in their interior, which renders treatment futile. The appearances of the exposed pulp always indicate alterations in the blood vessels, which are not irremediable, and toward the relief of which the treatment should be directed.

The capillaries are enlarged, the pulp at the point of exposure is often quite red, which redness passes away by gradual gradation into the normal grey tissue; some are exuding lymph, and more rarely pus.

It must, however, here be questioned, whether the term pulpitis, which has been used in connection with odontalgia, is a correct one. After a careful examination of very many pulps of the worst description, after the extraction of the aching tooth, the appearances have not indicated anything more than congestion, the pulp tissue being not reddened throughout, and at no point presenting the line of demarcation. A tooth with a normal sized foramen would appear to be incapable of maintaining its functions under the influence of active congestion. When the suffering of an attack of continuous pulp congestion has been endured for a time, the pain ceases, and afterwards the pulp is found to be devitalized by strangulation. I have not been able to come to any other conclusion than that the blood vessels of the pulp rarely pass beyond the stage of acute congestion, when its death occurs. It was my unexpected fortune once to examine a pulp which had a perfectly defined abscess within its parenchyma, and which presented every indication of having passed through all the stages of inflammation. The whole of the tissue was violently red, and the line of demarcation was purplish, and entirely surrounded a small collection of pus; the tooth was a large central incisor of an adult person. There existed, however, a condition which rendered the supply of blood sufficient to produce the described result. The foramen was a very large one, being probably one-twentieth of an inch in diameter.

TREATMENT.

As was said before, the treatment should be directed to relieve the congested state of the capillaries. This can only be done by a somewhat prolonged treatment. My practice is to define the borders of the cavity and remove the softer caries and fully expose the pulp to the air, if it be not already exposed. The immediate pain is relieved by local depletion, when practicable, by kreosote or caryophyllum, whichever seems applicable; then I close the cavity with tincture of aconite, beneath a cap of any substance which will protect from pressure. The cavity is filled with cotton, partially saturated with mastic or sandarac, and allowed to remain for several days. When, if the tooth has remained comparatively comfortable, it is more tightly closed, using Hill's stopping, or its equivalent, for an external protection, the dressing of aconite being applied, but not permitted to remain long in this condition, since the space between the arched cap and the pulp will fill with effused fluids. The coverings in direct contact with the pulp should be affected as soon as there is reason to believe the effusion has passed away. At this stage of the treatment I invariably, unlike in the previous classes, combine chemical with therapeutical action. The exposed surface is cauterized with pure carbolic acid, which is believed to produce a pellicle of coagulated albumen of a more acceptable nature to pulp tissue than any artificial covering which may be given. The immediate covering of the pulp I make of oxide of zinc and carbolic acid.

The cavity is now filled, every precaution being taken to prevent the least compression. Should the result be unfavorable the conclusion may be safely made in the present state of our knowledge of therapeutics, that the pulp is in an irremediable condition. I would relate here a remarkable case, which illustrates through how many and unfavorable vicissitudes a pulp may be ultimately saved, and protected at last by the deposit of secondary dentine.

*Case.*—Mesial surface of left lower inferior molar, exposed at both cornua.

Treated as usual, 1871. January 12th, 1872, the cavity was opened, when no apparent change in the appearance was observable. Aconite was applied, also dilute carbolic acid in glycerine; it was then carefully covered. There was, however, some sensation, as the external filling of gutta percha was introduced.

January 20th. The patient returned, having had some pain for three days, which was increasing. The dressings were removed, when the pulp bled. After the bleeding ceased aconite was applied, and afterwards pure carbolic acid; the pain was entirely relieved. (It should be mentioned, that in removing the gutta percha on January 12th, accidental pressure was made upon the pulp, which excited considerable pain).

January 29th. Patient reported no further trouble since seeing him.

October 12th, 1876. Opened and examined, when one corner was found entirely solidified; the other one was not disturbed. The case was then permanently filled, and has recently been examined and found vital.

The power of the pulp to protect itself by deposits of secondary dentine, or as it has been more properly designated, reparative dentine, has become an unquestionable fact, and now remains no longer a matter of doubt in the first, second and third class of disturbances.

It would appear from the accumulation of reliable experiences, that when there has not been disorganization of the dentinal cells of the periphery of the pulp, this result may be expected if the treatment is intelligently conducted and carried out by a hand capable of the necessary delicacy. In the last class, however, it is questionable whether this result ever takes place, and when we have had a broad exposure, which has been at last protected by secondary dentine, as has several times occurred in my experience, the inquiry may be fairly made, whether the diagnosis was not rather a topographical than a physical one, and that the case more properly belonged to the third class, notwithstanding the breadth of the surface exposed to irritating influences. It has been my experience to frequently uncover pulps which have tolerated the dressings for several years—in some cases over five—with entire comfort, the gutta percha coverings having presented no change in their appearance. In some cases the most careful handling has been again followed by considerable disturbance.

My practice now is, to fill such cases permanently, after several years of trial, if they remain nominally responsive to cold, without removing the coverings over the pulp.

It is gratifying to find that this system of treatment is extending, which gives promise that in the future a difficult and most painstaking operation will be rendered more reliable as its literature enlarges.—*Transactions Pennsylvania Dental Society.*

